# Conjugate and 23-valent pneumococcal polysaccharide booster vaccination in asplenic patients with thalassemia major: A randomized clinical trial study

**Published:** 2017

**Authors:** Mohammad Sadegh Rezai, Javad Ghaffari, Mohammadreza Mahdavi, Amir Bahari, Shahram Ala

**Affiliations:** 1 Infectious Diseases Research Center focus on Nosocomial Infection, Mazandaran University of Medical Sciences, Sari, Iran.; 2 Thalassemia Research Center, Mazandaran University of Medical Sciences, Sari, Iran.; 3 Mazandaran University of Medical Sciences, Sari, Iran.; 4 Pharmaceutical Sciences Research Center, Mazandaran University of Medical Sciences, Sari, Iran.

**Keywords:** PPV-23, PCV-13, Thalassemia Major, Asplenia

## Abstract

**Background:**

Pneumococcal vaccine provides protection against invasive pneumococcal disease in population at risk. This study was conducted to compare the antibody response to 13-valent pneumococcal conjugate vaccine and 23-valent pneumococcal polysaccharide vaccine in patients with thalassemia major.

**Methods:**

A randomized cross-over clinical trial was performed on 50 asplenic patients with thalassemia major who referred to thalassemia center at Bouali Sina Hospital, Sari, Iran from 2013 to 2014. Patients were divided into two equal groups. The first group received 13-valent pneumococcal conjugate vaccine (PCV) injected into the deltoid muscle at first and received 23-valent polysaccharide vaccine (PPV) by the same way two months later. The second group received PPV vaccine at first and PCV13 two months later. Levels of serum antibody were checked and measured by enzyme-linked immunosorbent assay (ELISA) before vaccination, and then 8 weeks after the first injection and 2 months after the second injection in all patients. Each time 0.5-ml dose of the vaccine was injected.

**Results:**

Of the 50 patients, three cases were excluded due to lack of cooperation and avoidance of vaccination. From 47 patient participants, 28 (59.6%) were males and 19 (40.4%) were females with age ranged between 20 to 44 years (average age of 29.6±1.4 years). Pneumococcal IgG levels in a group that used PCV before PPV (Group A) increased from 114.5±87.7 to 1049±720 U/ml (p=0.0001) and in another group that used PPV before PCV (Group B) increased from 115±182.2 to 1497.3±920.3 U/ml (P=0.0001).

**Conclusion:**

It can be concluded that PCV vaccine before PPV can be more effective in asplenic thalassemia major patients as a booster dose.

Thalassemia is a heterogeneous group of autosomal recessive genetic disorders due to failure in hemoglobin synthesis which can lead to anemia and ineffective erythropoiesis (1). In our country and similar other areas like the Mediterranean, North and West Africa, and the Middle East regions, thalassemia is generally common in the northern and southern provinces, as 10% of people at the Caspian coastline are carriers of the thalassemia gene. The global incidence of the disease is 2 per 1,000 live births (2, 3). Iron overload and splenectomy due to hypersplenism is usually performed because of some co-existing complications (4, 5). Morbidity in these patients occurs following heart failure, severe infections or as complications of splenectomy. Infection is the second leading cause of mortality after heart failure in these patients (3).

Some studies have shown that T cells, humoral immunity and phagocytes (phagocytosis defect due to tetrapeptide tuftsin deficiency) are impaired in these patients, and even in some cases, dysfunction of the complement system and opsonizasion and natural killer cells (NK) have also been reported (6). Ghaffari et al. showed no impairment of immunoglobulin levels in patients with thalassemia major (7). Organisms such as Yersinia enterocolitica, Klebsiella, E.coli, Streptococcus pneumoniae, Pseudomonas aeruginosa, Listeria monocytogenes and Legionella pneumophila cause infection in these patients that can be due to body iron overload (8-10).

In asplenic patients, infections such as meningitis, pneumonia, septicemia following contamination by encapsulated bacteria such as Streptococcus pneumoniae, Neisseria meningitidis, Hemophilus influenza type b are more common (11, 12). Pneumococcus is the most common pathogen (70%) that causes sepsis after splenectomy and is associated with 50% to 70% mortality rate (13).

Pneumococcal vaccine provides protection against invasive pneumococcal disease in people at risk. Various types of pneumococcal vaccines are used such as 23- valent polysaccharide vaccines (PPV), 7- valent conjugate pneumococcal vaccine and 13-valent pneumococcal conjugate vaccine (PCV). PCV vaccine compared to PPV is more effective for reduction of pneumococcal invasive disease because of more immunogenicity power and more stimulation of memory cell immunity, but covered a lower spectrum of pneumococcal strains. Combination vaccine is recommended for patients with sickle cell and HIV (11, 12). PPV is recommended for all asplenic children over two years old and 13- valent conjugate vaccine is recommended from two months old (12). In 2007, 7- valent conjugate pneumococcal vaccine (PVC, Prevnar) was licensed with USA get certified and caused dramatic decrease in invasive pneumococcal infection (13, 14). PCV13 was confirmed in preventing noninvasive pneumococcal disease and otitis media (12). Several studies have shown that the PPV vaccine administered following PCV has more immunogenic effect than PCV alone, therefore combining the application of PCV and PPV, the conjugate before polysaccharide is recommended for patients with AIDS and sickle cell (13).

Usually after 5-10 years of PPV vaccination, immune response may be reduced. Thus, a booster dose of vaccine may be needed. Pnuemovax booster should be considered every 5 to 10 years. There is insufficient evidence recommending PPV or PCV or both as boosters. To answer this question; which vaccine is better to inject before the other, we planned a crossover clinical trial to have a better protection using both vaccines. On the other hand, we could not find similar clinical trial. So the aim of this study was to compare the antibody response to 13-valent pneumococcal conjugate vaccine (PCV) and 23-valent pneumococcal polysaccharide vaccine (PPV) in asplenic patients with thalassemia major.

## Methods

A randomized double blind crossover clinical trial was performed on 50 asplenic patients with thalassemia major who referred to thalassemia center at Bouali Sina Hospital, Sari, Iran from 2013 -2014. The study was approved by the Research Ethics Committee of Mazandaran University of Medical sciences. Informed consent was obtained from each participant or parent/guardian. Inclusion criteria were all asplenic patients (no age limit) with thalassemia major who had received pneumococcal vaccine more than 5 years ago and their pneumococcal serum IgG level was less than 0.34 mg/dl. Those who received pneumococcal vaccine less than 5 years prior to enrollment in the study were excluded. According to statistical estimation and quantities of serum antibody level, the estimated number of sample case was 50 (25 cases in each group) based on 80% power and 95% accuracy. Demographic characteristics of the patients were recorded (table 1). Consequently, all of them had pneumococcal antibody less than normal before the start of vaccination.

The eligible patients were divided into two groups (A and B) based on sequential and random. Group A was vaccinated at first by PCV^13^ vaccine (Prevnar 13) manufactured by Wyeth Lederle Vaccines SA, Belgium] and then after 8 weeks, they received PPV vaccine (PSV23 polysaccharide vaccine manufactured by Sanofi Pasteur MSD AG 6340 Baar H4741.SZ, Switzerland) injected into the deltoid muscle. 

Group B patients received PPV vaccine at first and then PCV13 vaccine manufactured by the same companies 8 weeks later. Serum IgG levels were checked 8 weeks after the first dose of vaccine. Likewise, we checked serum antibody levels after 8 weeks of second vaccine in both groups. Serum samples were obtained before each immunization and 8 weeks after second immunization. We used 0.5-ml dose of the vaccine. The IgG antipneumococcal antibody levels of the patients were measured using VaccZyme Anti-PCP IgG Enzyme-linked Immunosorbent assay (ELISA) with a normal range of 3.3-270 mg/L. All samples were reserved in -20°^C^ before analysis. Adverse effects were assessed via an interview and physical examination. Subjects recorded reactions at 24 and 72 hours after each immunization. Fever was defined as oral temperature ≥38.5. We also recorded some local reactions including; redness, inflammation and pain in injection site. Analysis was done to determine the serum IgG improvement and alteration between the two groups.

Data were analyzed using SPSS software, Version 17, and descriptive statistical tests; Mean and standard deviation (SD), and Wilcoxon and Mann-Whitney tests. P<0.05 were assumed to be significant statistically.

**Table 1:** Classification of the both groups based on vaccine injection. Asplenic thalassemic patients (n=50)

**Group A**	**Group B**
Evaluation of IgG level before vaccination (n=25)	Evaluation of IgG level before vaccination (n=25)
PCV was injected	PPV was injected
Evaluation of IgG 8 weeks after first vaccine	Evaluation of IgG 8 weeks after first vaccine
PPV was injected	PCV was injected
Evaluation ofIgG 8 weeks after second vaccine	Evaluation ofIgG 8 weeks after second vaccine

Pneumococcal conjugate vaccine (PCV); Pneumococcal polysaccharide vaccine (PPV); Immunoglobulin G (IgG)

## Results

Out of fifty patients enrolled in this study, 47 patients completed the study. Totally 28 (59.6%) patients were males and 19 (40.4%) were females. The age range of the participants was from 20 to 44 years old with the mean age of 29.6±1.4 years. All participants underwent splenectomy with an average time of 15.2±5.2 years passed from the operation and a minimum and maximum of 6 to 26 years from the beginning of this research respectively. Three patients were excluded; two died due to thalassemia and co-existing disease and one because of dermatologic problem.The antibody level of the all participants was measured before and after intervention (table 2). Comparison of IgG antibody levels before and after the first vaccine was not significant (P=0.2 and 0.7, respectively) but is significant after the second vaccine (P=0.049). Immunologic response in group A who initially received PCV vaccine before PPV was significantly higher than group B after the second injection (P=0.049). All of them had pneumococcal serum IgG more than 0.34 mg/dl in which a cutoff level is considerable for prevention of possible infection after splenectomy. Of course change in serum level of IgG is more important for us than the absolute cutoff level.

**Table 2:** Comparison of IgG antibody (mg / L) in both groups before and after intervention

**Groups**		**Number**	**Mean±SD**	**P-value**
Group A	Before intervention (group A) After first vaccine PCV(group A)	23 23	114.5±87.7 844±872.4	0.0001
	Before intervention (group A) After PPV (group A)	23 21	114.5±87.7 1049±720.3	0.0001
Group B	Before intervention (group B) After first vaccine PPV (group B)	24 24	115±182.2 1089.8±979	0.0001
	Before intervention (group B) After second PCV (group B)	24 22	115±182.2 1497.3±920.3	0.0001

Pneumococcal conjugate vaccine (PCV); Pneumococcal polysaccharide vaccine (PPV)

## Discussion

So far as we have known this is the first study to evaluate the effect of PCV13 in asplenic patients with thalassemia major. Gram-positive Streptococcus cocci pneumonia is the most common cause of community-acquired pneumonia, and the second cause of bacterial meningitis, and the common cause of bacteremia (14). Pneumococcus is the most common pathogen responsible for postsplenectomy sepsis associated with 50 to 70 percent mortality (11). Meningitis caused by this germ is associated with 10% mortality and 20 % to 30% hearing loss. Neurological complications of this organism included epilepsy, blindness, paralysis and decrease in IQ (15). Resistance of this germ to antibiotics is a major health problem, as 15-30 percent of the isolated microbes are resistant to 3 or more antibiotics called multidrug resistance (MDR). 

Pneumococcal vaccine induces protection in individual at risk against invasive pneumococcal disease. We found a significant difference in the antibody levels after receiving the initial vaccine (PCV or PPV) between the two studied groups. Our findings were similar to others. (12, 15, 16). This significant statistical difference represents the efficacy and immunogenicity of each vaccine. However, no significant difference was observed between the first and the second injections, but finally after the third injection the antibody levels became significantly higher in group A that received PCV at first. Similar to our study, George Tupolousus et al. in Greek performed a study on asplenic thalassemia patients. 

They divided the patients into two studied groups. The first group received PCV13 at first and PPV23 after one month. The second group received two PPV with one-month interval. Then both groups received PPV after one year. The control group received PCV and PPV one year later. In both groups without splenectomy, the effect of both methods on antibody levels was similar. Injection of PPV had no significant effect, but frequent injection of PCV caused decreased in some pneumococcal strains among asplenic patients (12).

A study showed that PCV can overcome the decrease of PPV response, but our result showed that this theory is not true in all circumstances, and the responses of patients to the vaccines did not have significant difference in both groups in the short term but may be accurate for the long-term (17). Unlike the previous studies, our study showed that the injection of PCV before PPV has no effect on the immunogenicity of PCV the short-term because all of our patients in both groups had antibody level higher than the threshold of 0.34 μ gr/dl after the first dose of antibody (10, 18). As a consequence, whether the PCV is injected first or after injection of PPV, both methods are equally effective in the short term and can prevent invasive pneumococcal diseases. Nonetheless, in one study on asplenic thalassemia patients, PPV injection after PCV in reducing invasive pneumococcal disease was more effective (19). In contrast, another study showed that when PPV was injected alone or before the PCV, it was associated with lower antibody response (20). An important difference of the result of our study with others was that we used PCV13 instead of PCV7.

Although there are controversies related to conjugate and polysaccharide vaccines about the effect of age on efficacy of the vaccines, in our study, because the age range of our patients was between 20 to 44 years, we could not conclude the age effect on the efficacy of the vaccines. (20, 21).

Louis Verrnachivou et al. conducted a survey on patients with sickle cell anemia. They divided the patients into two groups. One group received PPV vaccine and the other group received PCV in two months. At the start of the study, all participants received Hemophilus conjugate vaccine - tetanus and 4 valance meningococcal vaccines. Antibody level was checked by ELISA method before PPV vaccination and 3 to 6 weeks after PPV vaccine. Finally, antibody response was higher in combined method (both PPV and PCV injection) without more serious side effects (11).

In a study by Marveled Aging et al. performed on 130 subjects in Netherlands, serum antibodies against Streptococcus pneumoniae were evaluated in asplenic individuals or those who had splenic dysfunction. One hundred thirty individuals were vaccinated in the past 5 years. Antibody levels above the threshold 35.0 mg/dl were seen in 83 cases (64%) (22).

The result of a study conducted by Lauri A. Hicks in the United States from 1998 to 2004 demonstrated that the rate of pneumococcal disease caused by strains uncovered by PCV vaccination is increasing (14). Due to the risk and complications associated with the disease, it appears that more attention is needed regarding the serotypes and performing vaccination in these patients. Yet, PPV23 is routinely used to cover other serotypes, but the vaccine contains purified polysaccharide pneumococcal serotype 23, which includes more than 95% of this germ, more effects of these vaccine have been debated (15, 23, 16). In spito of that a population study on 870,000 individuals confirmed that PPV vaccination reduces hospitalization and pneumococcal respiratory disease (15). Of course, several studies reported skin reaction at the vaccine inoculation site as complication of vaccination that has been seen after the second injection (24). Comparison of group of patients who received only one dose of PPV vaccination with another group who received only one dose of PCV showed no significant difference. In David Goldblatt’s study, the total rate of IgG was equal among the groups that was similar to the present study. Even though, the dose of PCV is similar to another study (25), there is still controversy using PCV or PPV separately in different studies, but combination therapy has also been recommended (16). Similarly, Grimprel et al. of France in 2009, performed a study and their results emphasized this finding (26) A study by Jaime L. Rubine in the United States determined that in addition to increasing the safety of the PCV13 against PPV, it reduced health care costs about $ 11.6 billion over 10 years (27).

In the present study, by investigating the two groups; A3 and B3, we found that the patients who received PPV after a primary dose of PCV had a higher immunity compared to patients who received PPV at first. It seems PPV injection following initial dose of PCV has a beneficial effect on PCV immunogenicity. Even the combined PCV/PPV vaccination program has been recommended for patients with sickle cell anemia and HIV (11). 

In George Artopolosus’ study splenectomy was performed on 58 patients (35 asplenic and 23 with spleen) were investigated. The first group had positive history of receiving PPV vaccine but the second group received no pneumococcal vaccine. Splenectomized group was divided into two groups; the first group received PPV one month after receiving PCV vaccine and the second group had two PCV inoculations after one month interval. Both groups received PPV after a year. The control group received PCV at first and PPV after a year. The efficacy of two methods was similar in both groups without splenectomy. All the same repeat PPV shot reduces some pneumococcal strains in splenectomized patients (11).

For all that this study proved the effect of repeated injection of PPV to reduce pneumococcal infection in splenectomized patients, in Lewis Stray-Pedersen’s study performed on patients with no history of splenectomy, the combined approach had the greatest effect (28, 10). Nonetheless, in our study, in the group receiving initial PCV, the final antibody response was significantly higher after the second injection PCV and this means higher immunogenicity of PCV provided following initial injection. Stray-Pederson in a study conducted on patients with ataxia-telangiectasia found that using PCV as the initial vaccine and then using PPV as a booster dose can prevent severe pneumococcal disease (28). Mortezvokouet et al. recommended pneumococcal, meningococcal and hemophilus influenza vaccination with booster dose to reduce infection in asplenic patients or splenic dysfunction. Though the vaccine protocols are alternated. Still many people still did not respond to the vaccine and further research is required to obtain a suitable method. In people with immune deficiency, weak response to the vaccine is more possible. Most of these studies recommended the repeat PPV shot after 5 years, despite that some of them advised to repeat the vaccine every 5 years, every 6 years or every 3-6 years (29).

In conclusions**, **our findings indicate that PCV before the PPV than PPV before PCV as a booster has higher effect in asplenic patients with thalassemia major. This research has also shown that combined schedules increase the immunity of individuals against pneumococcal disease and the antibody response showed a significant difference between the two groups.

One of the ;imitations of our study in addition to lack of groups receiving only PPV or PCV vaccine that caused better comparison on the effect of combined vaccine was the lack of laboratory facilities for the study of different strains presented in the vaccine. We suggest further studies with more samples in splenectomized and non-splenectomized thalassemia. 
